# Extended Pharmacopeial Characterization of Surfactant Aerosols Generated by a Customized eFlow Neos Nebulizer Delivered through Neonatal Nasal Prongs

**DOI:** 10.3390/pharmaceutics12040319

**Published:** 2020-04-02

**Authors:** Federico Bianco, Elena Pasini, Marcello Nutini, Xabier Murgia, Carolin Stoeckl, Martin Schlun, Uwe Hetzer, Sauro Bonelli, Marta Lombardini, Ilaria Milesi, Marisa Pertile, Stephan Minocchieri, Fabrizio Salomone, Albert Bucholski

**Affiliations:** 1 Department of Preclinical Pharmacology, R&D, Chiesi Farmaceutici S.p.A., 43122 Parma, Italy; e.pasini@chiesi.com (E.P.); m.nutini@chiesi.com (M.N.); S.bonelli@chiesi.com (S.B.); m.lombardini@chiesi.com (M.L.); i.milesi@chiesi.com (I.M.); m.pertile@chiesi.com (M.P.); f.salomone@chiesi.com (F.S.); 2 Scientific Consultancy, 48640 Bilbao, Spain; xabi_murgia@hotmail.com; 3 PARI Pharma GmbH, 82319 Starnberg, Germany; Carolin.Stoeckl@pari.com (C.S.); Martin.Schlun@pari.com (M.S.); Uwe.Hetzer@pari.com (U.H.); Albert.Bucholski@pari.com (A.B.); 4 Division of Neonatology, Cantonal Hospital Winterthur, 8401 Winterthur, Switzerland; Stefan.Minocchieri@ksw.ch

**Keywords:** eFlow nebulizer, nebulized surfactant, aerosol deposition, non-invasive ventilation, nasal prongs

## Abstract

The delivery of nebulized medications to preterm infants during Non-Invasive Ventilation (NIV) remains an unmet clinical need. In this regard, the effective delivery of nebulized surfactant has been particularly investigated in preclinical and clinical studies. In this work, we investigated the feasibility of delivering nebulized surfactant through various commercially available nasal prong types. We first performed a compendial characterization of surfactant aerosols generated by the eFlow Neos nebulizer, customized to be used in neonates, determining the amount of surfactant delivered by the device as well as the aerodynamic characteristics of surfactant aerosols. Additionally, we extended the compendial characterization by testing the effect of different nasal prong types on the estimated lung dose using a realistic Continuous Positive Airway Pressure (CPAP) circuit that included a cast of the upper airways of a preterm neonate. The compendial characterization of surfactant aerosols delivered through different nasal prongs achieved relatively high delivered surfactant doses (in the range 63–74% of the nominal dose), with aerodynamic characteristics displaying mass median aerodynamic diameters ranging between 2.52 and 2.81 µm. Nevertheless, when using a representative in vitro setup mimicking NIV in a clinical setting, significant differences were observed in terms of the estimated lung dose accounting for up to two-fold differences (from 10% to 20% estimated lung deposition of the nominal dose) depending on the chosen nasal prong type. Considering that surfactant lung deposition rates are correlated with therapeutic efficacy, this study points out the relevance of choosing the appropriate NIV interface to maximize the lung dose of nebulized medications.

## 1. Introduction

The lungs of very preterm neonates are immature and fragile at birth, predisposing them to suffer from Respiratory Distress Syndrome (RDS). In many cases, RDS is followed by an intrapulmonary inflammatory state that induces the arrest of lung development and evolves to chronic lung disease [1], also termed Bronchopulmonary Dysplasia (BPD) [2,3]. The development of BPD may be involuntarily favoured by the life-saving intensive treatments required to revert the primary RDS, such as oxygen therapy and mechanical ventilation through a tracheal tube [4,5]. These treatments are well-known factors inducing lung inflammation. Therefore, modern neonatal intensive care tries to stabilize the lungs of RDS-infants with lower oxygen targets [6] and prioritize the use of Non-Invasive Ventilation (NIV) in order to reduce the exposure of preterm neonates to mechanical ventilation [7].

Over the last three decades, topical pharmacological interventions with exogenous surfactant have been demonstrated to be very effective in restoring the lung function of RDS infants [8]. The intratracheal delivery of exogenous surfactant neutralizes the high intrapulmonary surface tension, restoring gas exchange and improving lung mechanics. More recently, taking advantage of the good lung spreading properties of surfactant, Yeh et al. have demonstrated that supplementing a surfactant dose with budesonide reduces the incidence of BPD [9,10]. Unfortunately, the intratracheal delivery of surfactant is still considered an invasive procedure, which requires actual expertise to be performed during NIV (e.g., with a laryngoscope during less invasive surfactant administration). In addition, drug delivery associated with surfactant instillation is limited by the number of surfactant doses required by the patient and represents a serious drawback for drugs potentially requiring repeated doses.

The use of aerosol medicine has been proposed as a drug delivery method to implement topical pharmacological interventions in the context of RDS and BPD [11]. Drug delivery directly to the lungs increases drug availability at the site of disease and reduces systemic exposure. For instance, clinical investigations have addressed the use of aerosolized corticosteroids for the prevention of BPD in intubated preterm neonates [12,13]. The feasibility of delivering nebulized surfactant during Continuous Positive Airway Pressure (CPAP) ventilation has also been addressed in several clinical studies [14,15,16,17,18], in the search for a truly non-invasive surfactant delivery method that could avoid intubation. Eventually, nebulized surfactant could also enable the co-delivery of other drug molecules, which would significantly benefit from the intrapulmonary spreading properties of exogenous surfactant [19,20]. Clinical studies with nebulized surfactant during CPAP have demonstrated the safety of this surfactant delivery method; nevertheless, its efficacy in preterm infants with severe RDS remains uncertain due to the low lung deposition rates observed in this patient population [21]. To date, the effective delivery of nebulized medications to preterm infants during NIV remains an unmet clinical need.

Vibrating-membrane nebulizers have demonstrated an improvement in lung deposition rates in neonatal animal models compared to traditional jet nebulizers [22]. They also reduce the amount of air added to the ventilation circuit and show a minimal residual volume of the nominal dose, which are appealing features in the context of surfactant nebulization. The United States Pharmacopeia (USP), in chapter <1601>, Products for Nebulization Characterization Tests, describe a sequence of experiments to be tested with each new drug–device combination in order to characterize the drug substance delivery and the aerodynamic assessment of nebulized aerosols [23]. This guideline provides a robust method to test the performance of the nebulizer device with a given drug for pediatric and adult use, but does not provide specifications for preterm neonates. Additionally, the compendial method does not consider key elements significantly impacting lung deposition during neonatal ventilation such as the airflow generated during NIV, the ventilation circuit, the airway geometry of preterm neonates, their breathing pattern, nor the NIV interface (e.g., nasal prongs) [11].

Several nasal prong types with different geometric designs are available in the market as NIV interfaces for preterm neonates. Differences in design provide distinctive airflow characteristics that have been associated with marked variations in resistance to airflow [24]. Such differences may as well have an impact on the deposition of surfactant aerosols and, ultimately, may affect the lung surfactant dose in a clinical setting. We therefore designed this observational study to investigate the feasibility of delivering nebulized surfactant through various commercially available prong types to test their potential to be used as NIV interfaces during aerosol therapy in preterm infants. For this purpose, we first determined the surfactant delivery rate and the aerosol aerodynamic characteristics through the nasal prongs according to USP standards. Additionally, we extended the compendial characterization by testing the effect of different nasal prong types on the estimated lung dose using a realistic CPAP circuit that included a cast of the upper airways of a preterm neonate.

## 2. Materials and Methods

### 2.1. Nebulizer Device, Surfactant Preparation, and Nasal Prong Types

Sixty independent eFlow Neos vibrating-membrane nebulizer heads (PARI Pharma, Starnberg, Germany), customized to neonatal standards, were used in the present study. The nebulizer was controlled by the eVent-Neos controller (PARI Pharma, Starnberg, Germany). This neonate-focused nebulizer has been miniaturized to fit appropriately into non-invasive neonatal ventilation circuits and can nebulize undiluted surfactant at relatively high rates [25].

All experiments were performed nebulizing the commercially available, animal-derived surfactant preparation *Poractant alfa* (Curosurf^®^, Chiesi Farmaceutici, Parma, Italy). This surfactant is a natural preparation, extracted from porcine lungs, containing almost exclusively polar lipids, in particular, phosphatidylcholine (PC, about 70% of the total phospholipid content), and about 1% of specific low molecular weight hydrophobic proteins SP-B and SP-C, at a phospholipid concentration of 80 mg/mL. *Poractant alfa* is routinely administered as an intratracheal bolus at a dose of 200 mg/kg.

**Figure 1:**
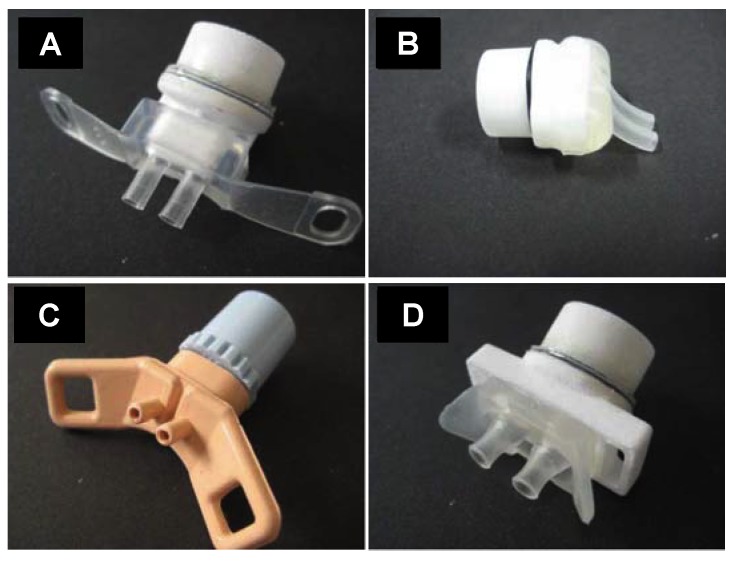
(**A**), Dräger XS prongs (Dräger); (**B**), Fisher and Paykel 3520 prongs (Fisher and Paykel Healthcare); (**C**), Argyle XS prongs (Cardinal Health); (**D**), Inspire prongs S (Inspiration Health). In (**A**,**B**,**D**) the prongs are connected to a customized, in-house-built white adaptor which allows the direct connection of the prongs to the nebulizer.

Four different nasal prong models with two different sizes of each type were used in this study (Supplementary Data 1): Dräger prongs (Dräger, sizes XS and L), Fisher and Paykel infant nasal prongs (Fisher and Paykel Healthcare; Ref: 3520 with 3.5 mm nare diameter; and Ref:5050 with 5.0 nare diameter), Argyle nasal cannula (Cardinal Health, sizes XS and L), and Inspire nasal prongs (Inspiration Health, sizes S and L). The prongs with the smallest size of each type are shown in Figure 1:

### 2.2. Compendial Breath Simulation Experiments to Determine the Delivered Dose

**Figure 2:**
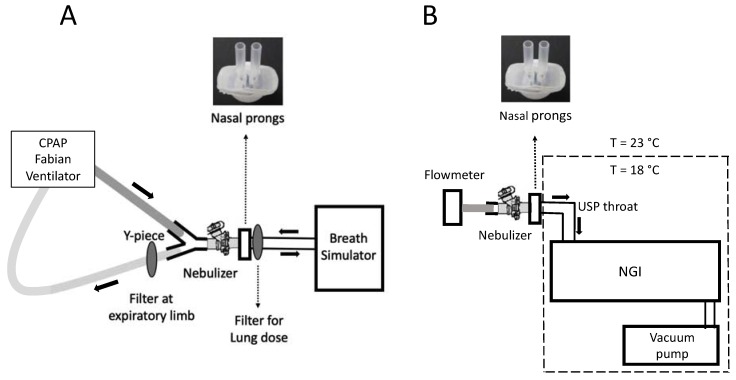
Scheme of the setups used for the pharmacopeial characterization of surfactant aerosols generated with the customized eFlow Neos and delivered through different types of nasal prongs. (**A**) describes the configuration for the breath simulation experiments and (**B**) describes the setup for the aerodynamic assessment of nebulized surfactant aerosols. Continuous Positive Airway Pressure (CPAP); United States Pharmacopeia (USP); Next Generation Impactor (NGI).

The delivered dose of nebulized surfactant was determined using the method described in the USP, general chapter <1601>, Products for Nebulization—Characterization Tests, adapted to the standards of preterm neonates. The in vitro setup is described in Figure 2A (Supplementary Figure S1). It consisted of a customized eFlow Neos nebulizer, nasal prongs as CPAP interfaces, one surfactant collection filter (PARI Filter PADs PZN: 00632160, Starnberg, Germany) placed immediately after the prongs to collect the delivered surfactant dose, another filter placed at the expiratory limb to estimate the amount of nebulized surfactant exiting the circuit, and a breath simulator. The breath simulator (Compas 2, PARI Pharma, Starnberg, Germany) was programmed with a sinusoidal waveform to deliver a tidal volume (VT) of 8.9 mL at a respiratory rate (RR) of 70 cycles/min and an inspiration:exhalation ratio of 1:1.5. The inspiratory arm was fed with an air-flow of 5 L/min to generate a CPAP of 5 cmH_2_O (Fabian HFO, Acutronic, Zug, Switzerland). The system was kept at a constant temperature of 37.0 ± 2.0 °C and relative humidity (RH) of 95 ± 5% (MR 730, Fisher & Paykel Healthcare). In each experiment, a total dose of 600 mg/kg of *Poractant alfa* (80 mg/mL) estimated for a preterm infant weighing 1.75 kg (13.125 mL or 1050 mg phospholipid of *Poractant alfa*) was delivered by the customized eFlow Neos nebulizer. After the delivery of each third of 600 mg/kg dose (4.4 mL or 352 mg phospholipid of *Poractant alfa*), the inspiratory and expiratory filters were replaced by fresh ones. The delivered surfactant dose was defined as the amount of surfactant recovered at the collection filter placed immediately after the nasal prongs. The experiments were performed in triplicate with each of the nasal prongs described above.

### 2.3. Next Generation Impactor (NGI) Experiments

The aerodynamic particle size distribution of surfactant aerosols was determined using the Next Generation Impactor (NGI, Copley Scientific, Nottingham, UK) coupled to the USP throat (induction port), as described in Figure 2B (Supplementary Figure S2). Both the USP throat model and the NGI were placed in a cooling chamber at a controlled temperature of 18 ± 0.5 °C. The nebulizer was connected to the nasal prongs, which were adapted to the USP throat using a customized rubber seal. The nebulizer and the prongs remained outside the tempered chamber at room temperature (23 ± 2 °C) and at relative humidity of 50 ± 5%. In each experiment, a volume of *Poractant alfa* of 3.3 mL (264 mg of phospholipid) was continuously nebulized. This amount of *Poractant alfa* was chosen because it avoids NGI cup overload and enables a straightforward phospholipid detection. The airflow of the impactor was set at 15 L/min. After nebulization, the amount of Poractant alfa remaining in the device, mouthpiece, induction port, and in all stages of the NGI was carefully extracted by rinsing each component with an organic solvent. This procedure was repeated with each nasal prong type in triplicate. For each case, the calculated delivered dose, fine particle fraction (FPF), Mass Median Aerodynamic Diameter (MMAD) and Geometric Standard Deviation (GSD) were determined.

### 2.4. Breath Simulation Experiments in a Realistic Neonatal CPAP Circuit

**Figure 3:**
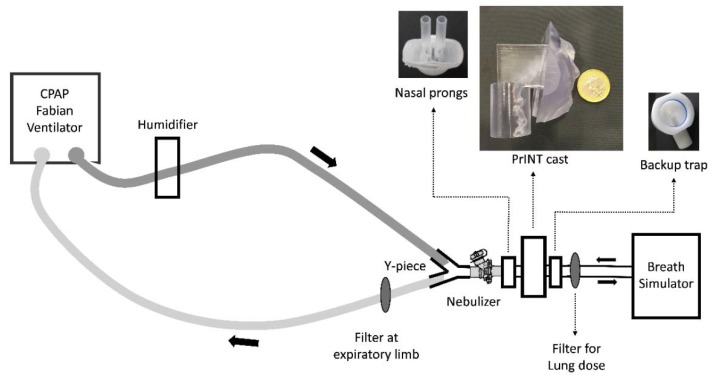
Scheme of the experimental setup of the breath simulation experiments in a realistic neonatal Continuous Positive Airway Pressure (CPAP) circuit.

The setup described above in the Compendial Breath Simulation Experiments section was further extended by including a cast of the upper airways of a premature infant (PrINT cast), yielding a representative in vitro setup mimicking NIV in a neonatal intensive care unit (Figure 3, Supplementary Figure S3) [11]. The airflow, humidity, temperature, and CPAP level of the setup, as well as the programmed breathing pattern, were the same as described in the previous section. The PrINT cast was digitally generated from the CT scans of a preterm infant born after 32 weeks of gestation, who had a birth weight of 1750 g [26]. The infant nose–throat cast was 3D printed (1zu1 prototypen, Dornibirn, Austria) as a solid substance (material: DSM ultra-clear water 10122). Surfactant collection filters were placed at the distal airway of the PrINT cast and the expiratory limb of the CPAP tubing. Also, a customized backup trap or surfactant collection cup was placed between the PrINT cast and the inspiratory filter to collect the nebulized surfactant that impacts against the walls after nebulization and moves forward as a liquid film. The surfactant collected at the filter placed at the distal airway of the PrINT cast technically represents the delivered lung dose. The prongs with the smallest size of each type were tested in triplicate in this setup (Dräger XS, Fisher and Paykel 3520, Argyle XS and, and Inspire S). The prongs were inserted in the nares of the PrINT cast and they were silicon-coated to achieve a tight fit. The surfactant dose, CPAP level, breathing pattern, circuit temperature and humidity were the same as previously described in the Compendial Breath Simulation Experiments section. Besides the amount of surfactant collected in the filters, the amounts of surfactant deposited within the CPAP circuit, backup trap, and PrINT cast were also determined. Before starting the experiments, the whole system was inspected for air-leaks.

### 2.5. HPLC Analytical Method for Phosphatidylcholine

A validated high-pressure liquid chromatography (HPLC) method with gradient elution using external standard calibration was used to determine the contents of PC in the surfactant collection filters and of all the components of the in vitro circuit, as previously described [25]. The method is sensitive enough to determine PC in the applied concentration range from 20–2100 µg/mL for Poractant alfa, provided that a dual-wavelength detector (WATERS 2487, Waters Corporation, Milford, MA, USA) is used.

### 2.6. Statistical Analysis

The data are presented as mean ± SD. One-way ANOVA with Bonferroni’s correction was used to analyze the datasets (IBM SPPS Statistics Software v23). A *p* < 0.05 was accepted to determine a statistically significant difference.

## 3. Results

### 3.1. Compendial Characterization of Surfactant Aerosols

The pharmacopeial characterization of surfactant aerosols generated with the customized eFlow Neos nebulizer through different nasal prongs is summarized in Table 1. Concerning the breath simulation experiments, the delivered dose ranged between 62% and 75% of the nominal surfactant dose (1050 mg of phospholipid), with the highest dose corresponding to surfactant aerosols generated through the smaller Dräger prongs (74.8 ± 0.7%). For Dräger, Argyle and Inspire nasal prongs, a higher delivered dose was achieved with the smaller size, also showing a lower amount of surfactant collected at the expiratory filter. Irrespective of the size, an equivalent delivered dose was observed with the Fisher and Paykel prongs, although a slightly higher surfactant amount was collected at the expiratory filter with the prongs of a bigger size.

The lowest mean nebulization time was observed using the Inspire S prongs, needing 64 min (range 51–71 min) to deliver 1050 mg of phospholipid of *Poractant alfa* (13.2 mL of undiluted surfactant). The highest mean nebulization was registered using the Argyle XS prongs (mean 127 min, range 90–175 min). Regardless of the prong type or size, the nebulization time showed noticeable variability across each of the replicates performed with the same prong type.

NGI experiments revealed a calculated delivered dose ranging between 84%, observed for the Fisher and Paykel 3520, and 91% for the Dräger S prongs. The lowest FPF was also found after nebulizing surfactant through the Fisher and Paykel 3520 prongs (62.8 ± 3.4%), whereas the highest was registered using Argyle L and Argyle XS prongs, with FPFs of 80.9 ± 1.2% and 81.7 ± 0.8, respectively. Regardless of the prong type or size, the MMAD was below 3 μm in every case. The GSD ranged between 1.53 and 1.64, except in the case of the Fisher and Paykel 5050 prongs, which showed a slightly higher mean value of 1.82.

### 3.2. Delivered Dose in A Realistic Neonatal CPAP Circuit

**Figure 4:**
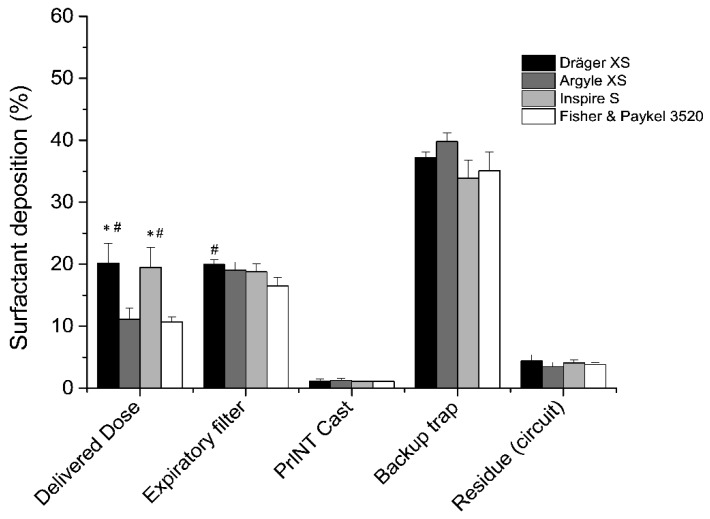
Mean cumulative percentage of deposited surfactant within different setup compartments using different types of nasal prongs. * *p* vs. Argyle XS and ^#^ *p* vs. Fisher and Paykel.

Placing a collection filter at the distal airway of the PrINT cast allowed us to estimate the theoretical lung dose of nebulized surfactant during realistic CPAP conditions. The highest delivered doses were registered when placing the Dräger XS (20.2 ± 3.2%) or Inspire S (19.5 ± 3.2%) prongs right after the eFlow Neos nebulizer, and were significantly higher (*p*<0.05) compared to the doses of nebulized surfactant delivered to the lungs using the Argyle XS (11.1 ± 1.8%) and Fisher and Paykel S (10.7 ± 1.1%) prongs (Figure 4). The amount of surfactant collected at the filter placed at the expiratory limb ranged between 15–20% of the delivered nominal dose. In this regard, a statistically significant difference was found between Dräger S and Fisher and Paykel 3520 prongs (*p* = 0.027). It is noteworthy that the highest proportion of deposited surfactant, accounting for over one-third of the nominal dose, was found in the backup trap or collecting cup that was placed between the PrINT cast and the inspiratory filter. The amount collected in the backup trap was the highest using the Argyle XS prongs (39.8 ± 1.4%), although no significant differences were registered compared to the other prong types. Irrespective of the prong type used, the amount of surfactant depositing within the PrINT cast (below 1.5% of the nominal dose) and the residual surfactant traces remaining in the circuit elements (below 5% of the nominal dose) were similar for all tested prong types.

**Figure 5:**
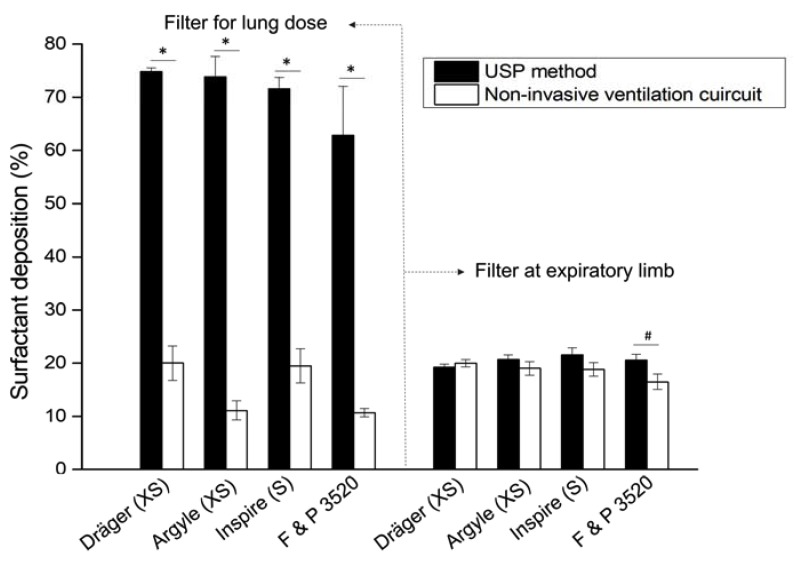
Mean delivered surfactant dose percentage (left) and mean surfactant percentage collected at the expiratory filter (right) after nebulization of surfactant with a customized eFlow Neos nebulizer through different nasal prong types. The method to estimate the delivered drug dose described in the United States Pharmacopeia (USP, solid bars) was compared to the drug dose delivered during realistic, neonatal non-invasive ventilation conditions. The values represent the percentages of a nominal dose of 1050 mg of phospholipid of undiluted Poractant alfa. * *p* < 0.0001 and ^#^ *p* < 0.01.

If the compendial method and the realistic neonatal ventilation circuit are compared, significant differences in terms of the delivered dose can be observed in all cases (*p* < 0.001, Figure 5), without marked differences in the amount of surfactant collected at the expiratory filter. Exceptionally, a marginal significant difference was detected in the surfactant amount collected at the expiratory filter for the Fisher and Paykel 3520 prongs (*p* < 0.01).

## 4. Discussion

This study demonstrates the feasibility of delivering surfactant aerosols generated with the customized eFlow Neos nebulizer through a selection of commercially available nasal prongs. The compendial characterization of surfactant aerosols delivered through different nasal prongs achieved relatively high delivered doses (range 63–74% of the nominal dose), with aerodynamic characteristics displaying MMADs ranging between 2.52 and 2.81 µm. Nevertheless, the extended characterization of surfactant deposition using a representative in vitro setup of NIV revealed significant differences between different nasal prong designs in terms of the estimated lung dose, which accounted for up to two-fold differences (from 10% to 20% of the nominal dose) depending on the chosen nasal prong type.

Aerosol delivery of medications to preterm infants, while they are managed with NIV, has been attempted for decades with limited success [11,27]. Eventually, an efficient method of non-invasive aerosol delivery should enable the safe prevention and treatment of lung conditions associated to preterm birth, such as RDS and BPD, by the topical administration of nebulized surfactant, anti-inflammatory drugs, and other molecules that promote lung development [27,28]. Unfortunately, lung deposition in preterm infants is very low, and is fundamentally affected by the low lung volumes and fast breathing rates of preterm babies, the nebulizer type, the diluting effect of the bias flow during NIV and the NIV ventilation circuit, including the NIV interface [11,29]. While the intrinsic characteristic of preterm infants that limit lung deposition cannot be changed, the right choice of nebulizer type, NIV method and its settings, and the appropriate NIV interface may contribute to increasing lung deposition in preterm infants.

In this study, aerosols from undiluted surfactant (*Poractant alfa* at 80 mg/mL) were generated with the eFlow Neos nebulizer. The first part of the compendial characterization consisted of determining the delivered dose and delivery rate of surfactant aerosols generated with the customized eFlow Neos through a collection of commercially available nasal prongs. For the breath simulation experiments, chapter <1601> of the USP indicates distinctive breathing patterns for adult, pediatric, and neonatal patients [23]. The neonatal breathing pattern provided in the USP mandates a sinusoidal waveform to deliver a *V*_T_ of 25 mL at a RR 40 cycles/minute and an inspiration:exhalation ratio of 1:3. In our opinion, these parameters do not consider the particular breathing pattern of preterm neonates with RDS. For instance, V_T_ is high and RR is rather low, which may overestimate the delivered surfactant dose under these testing conditions. Therefore, we adapted the method by programming a more accurate breathing pattern for a preterm infant (*V*_T_ = 8.9 mL, RR = 70 cycles/minute and an inspiration:exhalation ratio of 1:1.5). Moreover, the setup recommended by the USP was enhanced with a source of humidified air that generated a controlled CPAP of 5 cmH_2_O. Under these testing conditions, the amount of nebulized surfactant passing through all prong types and collected at the filter that represents the delivered dose was relatively high (>60% of the nominal dose) in all cases. Applying the USP method, we did not find marked differences between different nasal prong designs or different sizes. Interestingly, in three out of four prong types, a higher delivered dose was found for the smaller size. In this regard, one could hypothesize that smaller prongs, with narrower sections, would promote the impaction of surfactant aerosol droplets into the walls of the prongs, thereby reducing the amount of surfactant aerosol droplets reaching the filter for the lung dose. Nevertheless, in the USP method, the aerosol droplets impacting against the prongs advance as a liquid film pushed by the CPAP flow and are captured in the filter for the lung dose. Therefore, the USP method provides robust data on the overall surfactant amount existing in the nebulizer–nasal prongs combination, but it does not provide an accurate estimation of the surfactant that reaches the filter as aerosol particles.

In order to estimate the lung surfactant dose that may eventually reach the lungs of preterm infants, we extended the in vitro setup by including a cast of the upper airways constructed from the CT scans of a preterm neonate [26] and a backup trap that collected the fraction of surfactant aerosol impacting against the walls of the nasal prongs and the PrINT cast and moved forward as a liquid film. This realistic setup allowed us to discern between the overall surfactant delivered by the nebulizer through the nasal prongs and the actual surfactant fraction reaching the distal airways of the cast. Compared to the delivered dose determined with the compendial method, the lung dose was dramatically reduced in the extended setup. Irrespective of the used prong type, the amount of surfactant collected within the backup trap accounted for over one third of the nominal dose. This has relevant clinical implications because placing the nebulizer between the Y-piece and the nasal prongs will unavoidably generate a liquid surfactant film at the upper airway level.

We tested four different nasal prong designs in the extended setup. For these experiments, we chose the prongs with the smallest size because these fit better to very preterm infants, who have a higher probability of developing RDS and therefore might benefit the most from nebulized surfactant therapy. The lung doses achieved with different nasal prong designs ranged between 10–20% of the nominal dose and are relatively high compared to the historical lung deposition reported for premature infants (~1%) [21,30]. The surfactant lung dose estimates determined with our in vitro setup correlate well with some recent studies of surfactant lung deposition performed in animals. Gregory et al. found a surfactant lung deposition of 11.4% in non-human primates (4.8–6.8 kg), managed with a CPAP of 5 cmH_2_O after delivering radiolabeled Lucinactant aerosols generated with a capillary aerosol generator through nasal prongs [31]. Linner et al. reported lung depositions of 15.9% of technetium-labeled surfactant (nominal dose 200 mg/kg) in healthy neonatal piglets (1.2–2.2 kg) managed with nasal CPAP (3 cmH_2_O) delivered with customized nasal prongs [32]. Interestingly, the Linner study was conducted with the eFlow Neos nebulizer, the same nebulizer device used in the present study. The correlation between these in vivo data and our in vitro study shows the value of realistic in vitro neonatal ventilation setups as a tool to reduce animal experiments in the context of aerosol testing.

Dräger and Inspire prongs achieved significantly higher fractions of lung dose estimates (~20% of the nominal dose) compared to Fisher and Paykel and Argyle prongs (~10%). These differences are probably due to the different geometries of the nasal prongs. Dräger and Inspire prongs have straight designs, whereas Fisher and Paykel prongs are bent and display an angle which might promote surfactant impaction in the prongs’ walls. In the case of the Argyle prongs, even though they also have a straight design, they have a narrow section which increases the resistance to airflow and may also promote the impaction of bigger surfactant particles serving as a filter for smaller particles. Indeed, Argyle prongs showed the highest FPF (>80%). We would like to acknowledge that our aim in this work was to conduct an observational study of surfactant aerosols generated through different commercially available nasal prongs. Therefore, the reason for these significant differences observed with different nasal prong geometries is unknown to us and remains speculative.

We chose a nominal dose of 600 mg/kg of *Poractant alfa*, which is three-fold higher than the routine clinical dose used in instillation protocols [7]. This relatively high nominal dose was primarily chosen based on a theoretically expected lung deposition of 10%, which would ultimately yield a total lung surfactant dose of 60 mg/kg, a value that is in line with the surfactant pools reported in the literature for premature neonates [33]. Unpredictably, we found marked variations across individual experiments with regard to the nebulization time required to deliver the whole surfactant dose. It has previously been discussed that complex suspensions, such as surfactant formulations, could clog the pores of the vibrating mesh of the nebulizer [34]. However, we have recently demonstrated the consistent performance of the eFlow Neos in nebulizing *Poractant alfa*, achieving constant nebulization times after continuously delivering the same surfactant dose as reported in this study [35]. Nevertheless, in our previous study, surfactant aerosols were generated towards the open air, in the framework of laser diffraction experiments, whereas, in this study, surfactant aerosols were generated in a tightly sealed neonatal ventilation circuit. Therefore, we speculate that the observed differences in terms of nebulization time across different prong types are due to intra-circuit pressure variations mainly generated by the different prong geometries, which might have, in turn, affected the performance of the nebulizer device. Additionally, differences in nebulization time across replicates using the same prongs could be explained by slight differences in the circuit ensembling for each individual experiment. Anyhow, nebulization could significantly be reduced by using lower nominal doses. We have recently shown that a 600 mg/kg dose may promote surfactant accumulation in the airways of surfactant-depleted rabbits, yielding modest results in terms of gas exchange and lung mechanics [25]. Moreover, using the same device–surfactant combination as described here, a nominal dose of 200 mg/kg could revert the respiratory distress in surfactant-depleted neonatal piglets and adult rabbits achieving the best pulmonary outcomes with a nominal dose of 400 mg/kg of *Poractant alfa* [25,36].

## 5. Conclusions

In conclusion, this in vitro study proves the feasibility of delivering surfactant aerosols through a variety of commercially available nasal prong types to preterm neonates managed with NIV. The pharmacopeial characterization of the customized eFlow Neos Nebulizer using different prongs achieved relatively high delivered doses (>60% of the nominal dose), with MMADs below 3 μm with all tested nasal prongs. However, the extended characterization of surfactant aerosol deposition using a representative NIV circuit revealed two-fold differences in the estimated lung dose between different nasal prong types. These findings have relevant clinical implications since surfactant lung deposition rates are correlated with therapeutic efficacy. Therefore, we encourage the use of in vitro setups mimicking NIV in a clinical setting as a useful tool for aerosol deposition testing.

## Figures and Tables

**Table 1:** Summary of the compendial characterization of nebulized surfactant generated by a customized eFlow Neos nebulizer through different types of nasal prongs of different sizes.

Breath Simulation Experiments				Next Generation Impactor Experiments			
Nasal Cannula Type	Delivered Dose (%)	Surfactant at Expiratory Filter (%)	Nebulization Time (min)	Calculated Delivered Dose (%)	Fine Particle Fraction (%)	MMAD (µm)	GSD
Dräger prongs (S)	74.8 ± 0.7	19.2 ± 0.6	92 ± 36	91.1 ± 1.2	69.8 ± 4.6	2.81 ± 0.13	1.64 ± 0.10
Dräger prongs (L)	65.3 ± 0.6	23.5 ± 2.6	84.8 ± 11.7	86.3 ± 1.9	70.3 ± 4.1	2.81 ± 0.27	1.64 ± 0.11
Fisher and Paykel 3520	62.9 ± 9.2	20.6 ± 1.1	80.9 ± 28.5	84.1 ± 7.1	62.8 ± 3.4	2.53 ± 0.07	1.53 ± 0.05
Fisher and Paykel 5050	63.1 ± 1.2	23.3 ± 0.8	83.6 ± 25.4	86.3 ± 4.1	77.1 ± 8.7	2.76 ± 0.36	1.82 ± 0.46
Argyle (XS)	73.8 ± 3.9	20.7 ± 0.9	127.1 ± 43.59	88.0 ± 3.4	81.7 ± 0.8	2.79 ± 0.07	1.55 ± 0.04
Argyle (L)	71.7 ± 4.4	21.4 ± 0.2	85.1 ± 12.7	90.4 ± 2.9	80.9 ± 1.2	2.65 ± 0.02	1.53 ± 0.03
Inspire (S)	71.6 ± 2.1	21.6 ± 1.3	64.2 ± 11.58	90.4 ± 1	78.7 ± 5.4	2.75 ± 0.11	1.55 ± 0.06
Inspire (L)	65.5 ± 2.7	24.5 ± 2.5	112.8 ± 34.3	90.4 ± 1	74.2 ± 5.4	2.76 ± 0.12	1.58 ± 0.06

Mass Median Aerodynamic Diameter (MMAD); Geometric Standard Deviation (GSD).

## Supplementary Materials

The following are available online at https://www.mdpi.com/1999-4923/12/4/319/s1, Figure S1: The set-up for the compendial breath simulation experiments, Figure S2: The set-up of the compendial Next Generation Impactor (NGI) set-up for the aerodynamic assessment of nebulized surfactant aerosols, Figure S3: The set-up of the extended neonatal ventilation circuit designed for the breath simulation experiments.
