# Supplementary material for: Extended Pharmacopeial Characterization of Surfactant Aerosols Generated by a Customized eFlow Neos Nebulizer Delivered through Neonatal Nasal Prongs
Source: Pharmaceutics. 2020 Apr 2;12(4):319. doi: 10.3390/pharmaceutics12040319 (PMC7238214; doi:10.3390/pharmaceutics12040319)
Supplement: Supplementary file 1 [file pharmaceutics-12-00319-s001.pdf]

pharmaceutics

MDPI

Supplementary Materials: Extended Pharmacopeial Characterization of Surfactant Aerosols Generated by a Customized eFlow Neos Nebulizer Delivered through Neonatal Nasal Prongs

Federico Bianco, Elena Pasini, M Nutini, Xabier Murgia, Carolin Stoeckl, Martin Schlun, Uwe Hetzer, Sauro Bonelli, Marta Lombardini, Ilaria Milesi, Marisa Pertile, Stephan Minocchieri, Fabrizio Salomone, Albert Bucholski

Internal diameter (ID) of the nasal prongs used in the present study:

**Prongs type**	**ID (mm)**
Dräger prongs XS	n.a.
Dräger prongs S	n.a.
Fisher and Paykel infant nasal prongs 3520	3.5*
Fisher and Paykel infant nasal prongs 5050	5.0*
Argyle nasal cannula XS	n.a.
Argyle nasal cannula L	n.a.
Inspire nasal prongs S	3.3#
Inspire nasal prongs L	4.2#

*Provided by the manufacturer; #Own measurements; n.a. = ID not available.

Dräger prongs (Dräger); Fisher and Paykel infant nasal prongs (Fisher and Paykel Healthcare); Argyle nasal cannula (Cardinal Health); Inspire nasal prongs (Inspiration Health).

Supplementary Figure 1

Scheme and actual picture of the set-up for the compendial breath simulation experiments.

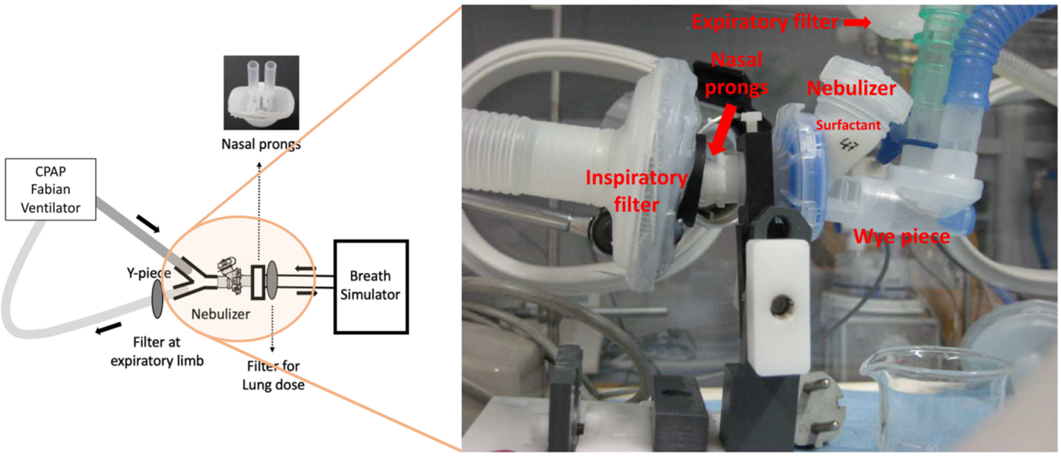

Figure S1. The set-up for the compendial breath simulation experiments.

Compendial breath simulation experiments to determine the delivered dose

The delivered dose of nebulized surfactant was determined using the method described in the USP, General Chapter <1601> Products for nebulization – characterization tests adapted to the standards of preterm neonates. The in vitro set-up is described in Figure 2A. It consisted of a

customized eFlow-Neos nebulizer, nasal prongs as CPAP interfaces, one surfactant collection filter (PARI Filter PADs PZN: 00632160, Starnberg, Germany) placed immediately after the prongs to collect the delivered surfactant dose, another filter placed at the expiratory limb to estimate the amount of nebulized surfactant exiting the circuit, and a breath simulator. The breath simulator (Compas 2, PARI Pharma, Starnberg, Germany) was programmed with a sinusoidal waveform to deliver a tidal volume (VT) of 8.9 mL at a respiratory rate (RR) of 70 cycles/minute and an inspiration:exhalation ratio of 1:1.5. The inspiratory arm was fed with an air-flow of 5 L/min to generate a CPAP of 5 cmH2O (Fabian HFO, Acutronic, Zug, Switzerland). The system was kept at a constant temperature of 37.0±2.0° C and relative humidity (RH) of 95%±5 (MR 730, Fisher & Paykel Healthcare).

Supplementary Figure 2

Scheme and actual picture of the set-up of the compendial Next Generation Impactor (NGI) set-up for the aerodynamic assessment of nebulized surfactant aerosols.

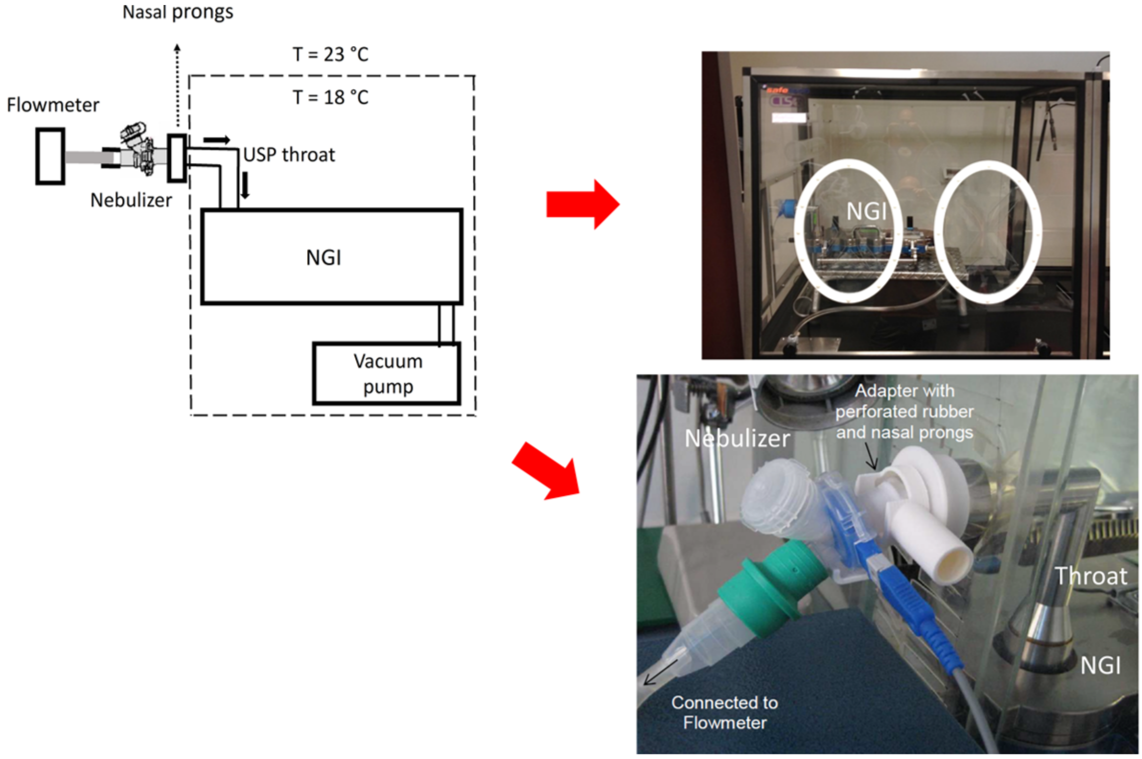

Figure S2. The set-up of the compendial Next Generation Impactor (NGI) set-up for the aerodynamic assessment of nebulized surfactant aerosols.

Next Generation Impactor (NGI) experiments

The aerodynamic particle size distribution of surfactant aerosols was determined using the Next Generation Impactor (NGI, Copley Scientific, Nottingham, UK) coupled to the USP throat (induction port), as described in **Figure 2B**. Both the USP throat model and the NGI were placed in a cooling chamber at a controlled temperature of 18 ± 0.5 °C. The nebulizer was connected to the nasal prongs, which were adapted to the USP throat using a customized rubber seal. The nebulizer and the prongs remained outside the tempered chamber at room temperature (23 ± 2 °C) and at relative humidity of 50 ± 5%. In each experiment, a volume of *Poractant alfa* of 3.3 mL (264 mg of phospholipid) was continuously nebulized. The airflow of the impactor was set at 15 L/min.

Supplementary Figure 3

Scheme and actual picture of the set-up of the extended neonatal ventilation circuit designed for the breath simulation experiments.

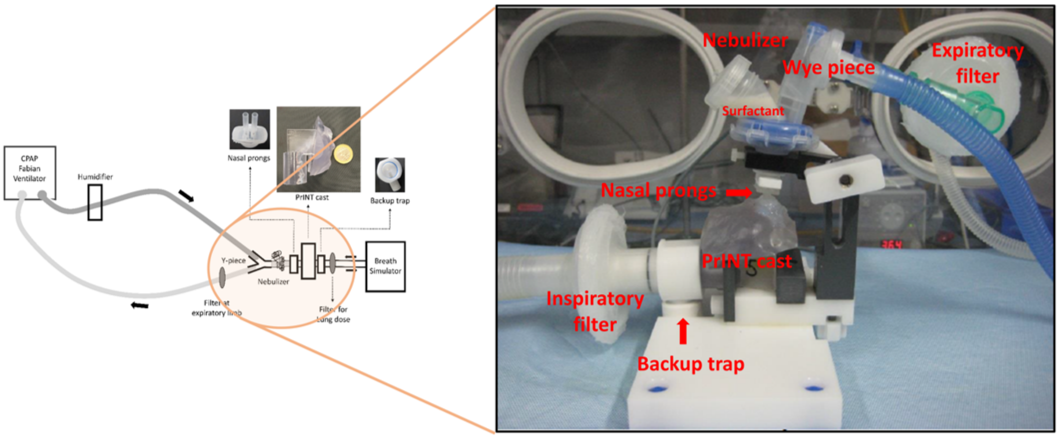

Figure S3. The set-up of the extended neonatal ventilation circuit designed for the breath simulation experiments.

Breath simulation experiments in a realistic neonatal CPAP circuit

The set-up described above in the Compendial breath simulation experiments section was further extended by including a cast of the upper airways of a premature infant (PrINT cast) and a backup trap placed between de PrINT cast and the inspiratory filter. The airflow, humidity, temperature, and CPAP level of the set-up, as well as the programmed breathing pattern were the same as described above.
